# Visible-near infrared spectral analysis for identification of physiological and genetic features in rice

**DOI:** 10.1270/jsbbs.25018

**Published:** 2025-10-07

**Authors:** Hinako Takehisa, Ichiro Nagaoka, Akifumi Ikehata, Yutaka Sato

**Affiliations:** 1 Institute of Crop Science, National Agriculture and Food Research Organization (NARO), 2-1-2 Kannondai, Tsukuba, Ibaraki 305-8518, Japan; 2 Institute of Fruit Tree and Tea Science, NARO, 2-1 Fujimoto, Tsukuba, Ibaraki 305-8605, Japan; 3 Center Region Agricultural Research Center, NARO, 1-2-1 Inada, Joetsu, Niigata 943-0193, Japan; 4 Institute of Food Research, NARO, 2-1-12 Kannondai, Tsukuba, Ibaraki 305-8642, Japan

**Keywords:** rice, spectroscopy, hyperspectral imaging

## Abstract

Visible-near infrared hyperspectral analysis is widely used for plant characterization and evaluation of agricultural products and food quality. On the other hand, it has remained un-certain whether this technique has a sufficient potential for evaluation of biological complexity during the growth of crop plants. In the present study, using a spectrometer and hyperspectral camera placed in a laboratory environment, we carried out continuous hyperspectral profiling of leaves derived from four rice cultivars grown under two field conditions. Combined analysis with transcriptome data revealed that the hyperspectral profile had potential to predict the degree of expression of developmentally regulated genes. In addition, principal component analysis of hyperspectral imaging data made it possible to detect growth-stage dependent dynamics and to distinguish differences between subspecies as well as field conditions by selecting an adequate pretreatment method. Furthermore, we obtained hyperspectral data for brown rice grains of recombinant inbred lines derived from a cultivar with high temperature tolerance during the ripening stage and with a good grain appearance. We then performed quantitative trait locus analysis using the extracted principal component scores and trait values related to grain appearance to explore the possibility of using spectral analysis for genetic studies.

## Introduction

Visible (VIS)-near infrared (NIR) hyperspectral analysis using a spectrometer and hyperspectral camera is a powerful tool for quality evaluation and content analysis in the field of agricultural and food science. In particular, NIR analysis is especially widely used for qualitative and quantitative assessment of biochemical components in various sample types such as solids, liquids, and powders based on chemometrics techniques. Recently, an increasing number of reports have documented the use of hyperspectral imaging to evaluate various plant traits (Sarić *et al.* 2022), such as nutrient elements (Bruning *et al.* 2019, Grieco *et al.* 2022, Herzig *et al.* 2019, Pandey *et al.* 2017, Pinit *et al.* 2022, Shi *et al.* 2024, Siedliska *et al.* 2021, Takehisa *et al.* 2022), disease severity (Feng *et al.* 2022, Lay *et al.* 2023, Lowe *et al.* 2017, Maina and Oerke 2024, Zhang *et al.* 2022), chlorophyll content (Banerjee *et al.* 2020, Yang *et al.* 2023), and grain properties (Barnaby *et al.* 2020). Hyperspectral imaging makes it possible to obtain hyperspectral data noninvasively, not only in the laboratory conditions but also under field conditions (Sarić *et al.* 2022). As environmental and genetic factors have an impact on crop production by affecting the growth and development in a morphological and physiological manner, it is important to efficiently evaluate the phenotypes of crop plants associated with quality and yield for increasing their potential. Although VIS-NIR hyperspectral analysis is a strong candidate for phenotyping, it would be difficult to connect spectral difference to what biological phenomena is detected and it has remained un-certain whether this technique has a potential for evaluation of biological complexity in sufficient depth.

We have performed comprehensive transcriptome profiling of rice plants under both field and laboratory conditions (Nagano *et al.* 2012, Sato *et al.* 2011, 2013, Takehisa *et al.* 2015), and developed nitrogen (N) and phosphorus (P) nutrient indicators to reveal the nutrient status dynamics associated with tiller development; rice plants express genes related to P acquisition and recycling in leaves during the tillering stages under field conditions with low available P, and their expression is rapidly suppressed in a dose dependent manner of N limitation (Takehisa and Sato 2019). Furthermore, we have indicated that P-indicator gene expression can be a useful index for early assessment of P status in rice plants, and predicted with high accuracy on the basis of VIS-NIR hyperspectral data obtained with a spectrometer and hyperspectral camera (Takehisa *et al.* 2022). Although transcriptome profiling has the highest resolution and yields the richest biological information, it is laborious and costly. Accordingly, combined use of hyperspectral analysis with transcriptome profiling is thought to have merit in compensating for the weakness of each approach.

Here, we performed hyperspectral profiling using VIS-NIR analysis of rice leaves in four cultivars under two field conditions throughout their entire growth and verified the potential of this approach for assessment of physiological state transition and genetic differences on the basis of our accumulated knowledge and by using transcriptome data that had been collected in our previous study (Takehisa *et al.* 2022). This potential was then further confirmed by hyperspectral imaging of rice leaves throughout the growth process. Furthermore, we collected spectral data for rice grains and performed quantitative trait locus (QTL) analysis using the extracted principal component scores and trait values related to grain appearance to explore the possibility of applying non-targeted spectral analysis to genetic studies.

## Materials and Methods

### Plant materials and cultivation conditions

Rice plants were cultivated at the National Agriculture and Food Research Organization (NARO) in Tsukuba (36°1ʹN, 140°6ʹE), Japan, during the normal cropping season. We grew 4 cultivars of *Oryza sativa* L. (*japonica*: ‘Nipponbare’ and ‘Koshihikari’, *indica*: ‘Takanari’ and ‘IR64’) under alluvial and andosol soil conditions in 2016 and 2020 for analysis during growth, and 10 *japonica* (‘Nipponbare’, ‘Koshihikari’, ‘Akitakomachi’, ‘Hitomebore’, ‘Sasanishiki’, ‘Toyomeki’, ‘Akidawara’, ‘Hinohikari’, ‘Mirenishiki’, and ‘Momiroman’) and 4 *indica* (‘Takanari’, ‘IR64’, ‘Habataki’, and ‘Hokuriku 193’) cultivars were used for analysis of flag leaves at the heading stage. In addition, for the QTL analysis we used recombinant inbred lines (RILs) derived from ‘Emi-no-kizuna’ and ‘Tomohonami’ (Nagaoka *et al.* 2019) cultivated in 2019. We measured traits related to days to heading (DH) and grain appearance (GA) related traits, i.e., percentages of perfect grains (PG), milky white grains (MW), basal white grains (BW), and white back grains (WB), using a grain quality inspector (RGQI90A; Satake Co., Ltd., Hiroshima, Japan).

### VIS-NIR reflectance spectroscopy

We collected uppermost expanded leaves from the four cultivars in 2016 and rice grains from 96 RILs selected on the basis of DH with scores similar to ‘Emi-no-kizuna’ and ‘Tomohonami’. Frozen leaves and brown rice grains were ground into powder using a Shake Master Auto BMS-A20TP homogenizer (Bio Medical Science). As was the case in our previous study (Takehisa *et al.* 2022), the leaf samples were dried at 60°C for 48 h to avoid detecting strong absorption patterns of water in the near infrared region. Approximately 50 mg of each sample was placed in a screw tube bottle and centrifuged at 3,000 rpm for 2 min. For intact brown rice grains, an appropriate number were placed in the bottle. The spectral reflectance of the samples was then measured between 400 nm and 2499.5 nm at 0.5-nm intervals using an XDS Rapid Content Analyzer (FOSS). Reflectance (R) was converted to absorbance (A) (A = log1/R).

### Hyperspectral imaging

We used an NH-7 (EBA JAPAN Co., Ltd.) covering the 350–1100 nm range with 151 bands and an SIS-I (EBA JAPAN Co., Ltd.) covering 900–1700 nm with 81 bands, in accordance with our previous study (Takehisa *et al.* 2022). As the light source, two halogen lamps were used for each system (300-W for NH-7 and 150-W for SIS-I). Leaf samples were placed on a light exclusion sheet (Super Black IR, Shibuya Optical Co., Ltd.) and scanned to finally acquire reflectance spectrographs in the visible (VIS) and near infrared (NIR) region (450–950 nm) with the NH-7 and the NIR region (950–1650 nm) with the SIS-I. In order to reduce the influence of light and noise in the raw hyperspectral images (Ir) and to obtain corrected reflectance images (Ic), white and dark corrections were performed using a white reference image (Iw) and a dark reference image (Id). The Iw was acquired using a white board (Color Checker White Balance, X-rite) and the Id by covering the camera lens with an opaque cap. The Ic was obtained using the following equation:


Ic=(Ir−Id)/(Iw−Id)

For extraction of a region of interest (ROI) from the VIS_NIR (450–950 nm) and NIR (950–1650 nm) imaging data, the spectral reflectance at 780 nm, and at 1260 nm and 1290 nm was used, respectively. All reflectance in the ROI was averaged and used as the reflectance spectrum of each sample.

### Analysis of hyperspectral data

We pretreated the spectral data by smoothing based on moving averages and the 1st and 2nd order Savitzky-Golay derivatives (window size = 11) with or without the standard normal variate (SNV). In addition, mean-centering was applied to the data with or without scaling, finally providing 12 pretreated data sets, i.e., smoothing, smoothing + scaling, SG1, SG1 + scaling, SG2, SG2 + scaling, SNV + smoothing, SNV + smoothing + scaling, SNV + SG1, SNV + SG1 + scaling, SNV + SG2, and SNV + SG2 + scaling. We used the partial least squares regression (PLS-R) for development of the prediction model. In addition, we calculated the normalized difference spectral index (NDSI) with Ri and Rj, the reflectance values at i and j nm wavelengths, as follows:


NDSI(Ri,Rj)=(Rj−Ri)/(Rj+Ri)

These procedures were performed in the R statistical environment with the “pls” and “signal” packages (R Core Team 2024). Principal component analysis (PCA) was conducted using the R program with the “prcomp” package. In order to evaluate the potential for prediction with hyperspectral data, we used the averaged gene expression values for each of the 54 clusters, which was reported in our previous study (Takehisa *et al.* 2022).

### QTL analysis

We used the “qtl” package in the R statistical environment for estimation of the linkage map and QTL mapping (Broman *et al.* 2003). For this analysis, we used 175 single nucleotide polymorphism markers (Nagaoka *et al.* 2017, 2019). Estimation of the linkage map was conducted with the Kosambi function setting. For QTL analysis, we used the 96 RILs based on DH and performed simple interval mapping with the EM algorithm. A threshold level of 5% for statistical significance was determined by 1000 permutations. We extracted QTLs with LOD score above the threshold level.

## Results

### Reflectance spectroscopy profiling of rice leaves throughout the growth process

**Fig. 1.**
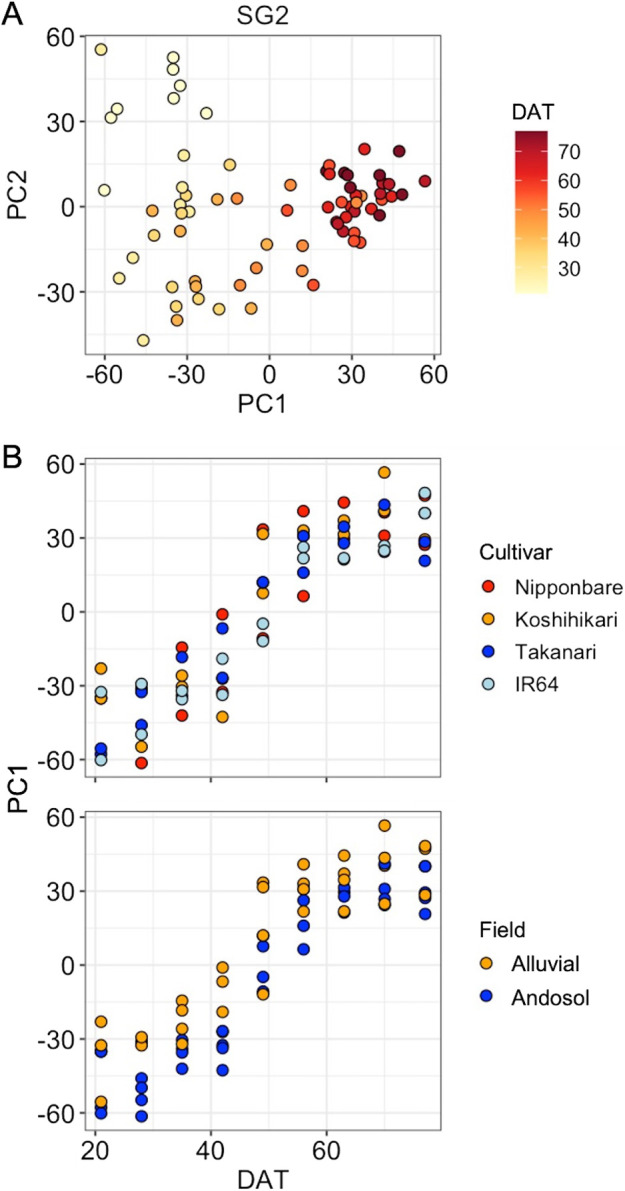
PCA of reflectance spectroscopy data from the four rice cultivars at nine time points from 21 to 77 DAT under two field conditions. (A) PCA with SG2 pretreated data. Color scale of plots is based on DAT. (B) Profiles of PC1 values throughout the growth process. Colors of plots indicate cultivars (upper) and fields (lower), respectively.

We obtained 72 sets of hyperspectral data using reflectance spectroscopy (400–2499.5 nm) from the four rice cultivars at nine points from 21 to 77 days after transplanting (DAT) under alluvial and andosol soil conditions in 2016 (Table 1). PCA was applied to three sets of pretreated data (smoothing, SG1, and SG2), and this revealed the growth-stage dependent differences most clearly in SG2 (Fig. 1A). Profiles of PC1 scores along the growth direction of the rice plants showed that although there was no variation in profiles among the cultivars, there tended to be differences between alluvial and andosol soil conditions at the early stage of growth (28, 35, and 42 DATs) (Fig. 1B).

**Fig. 2.**
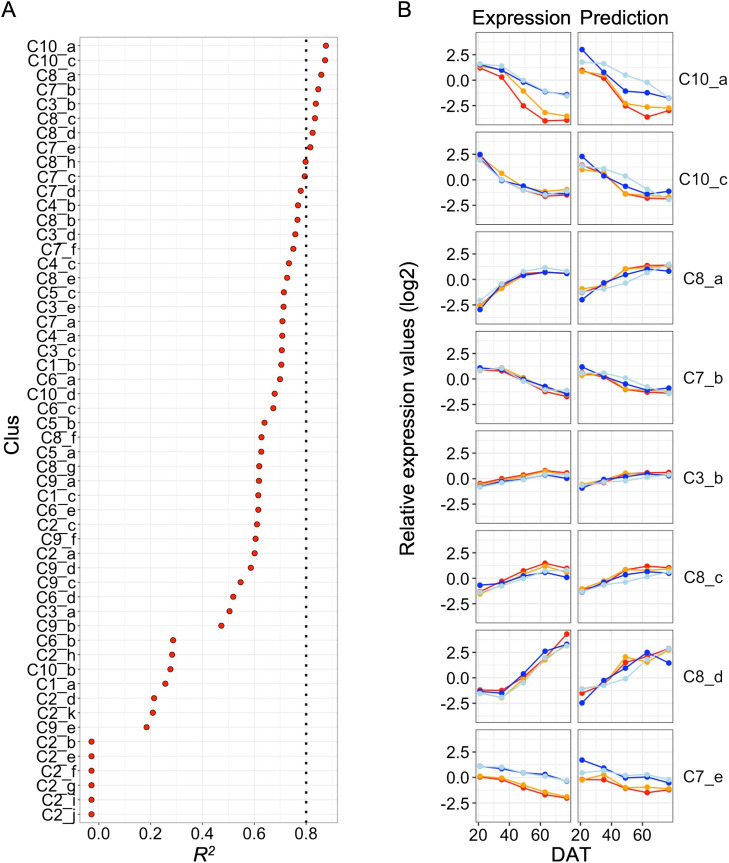
Evaluation of the prediction models with SG2 + scaling pretreated data. (A) Coefficient of determination (*R*^2^) of the prediction model relative to averaged expression values in the 54 clusters. (B) Comparison between the expression and predicted values in eight clusters with *R*^2^ values above 0.8. Red, orange, blue, and light blue lines show Nipponbare, Koshihikari, Takanari, and IR64, respectively.

In order to further evaluate the potential of reflectance spectroscopy to detect physiological and genetical differences, we attempted to predict transcriptome profiles using hyperspectral data. The transcriptome profiles consisted of 80 microarray data, which is derived from the four cultivars at 10 points from 13 to 77 DAT under alluvial and andosol soil conditions in 2016, and we divided 11817 genes into 54 clusters, e.g., Clus 1_a, Clus 1_b, and Clus 1_c for Clus 1, based on the similarities in expression pattern (Takehisa *et al.* 2022). Of note is that samples for the transcriptome profile and the reflectance spectroscopy were derived from leaves of rice plants at the same time and in the same field in 2016. In this study, we used 72 microarray data except for 13 DAT. We predicted the averaged expression level of the genes in each cluster with the PLS-R method using the 12 pretreated data and evaluated the prediction models with coefficient of determination (*R*^2^) by leave-one-out cross validation. Overall, SG2 + scaling tended to show the best performance in the prediction (Supplemental Fig. 1) and the *R*^2^ values of eight clusters, i.e., Clus 10_a, Clus 10_c, Clus 8_a, Clus 7_b, Clus 3_b, Clus 8_c, Clus 8_d, and Clus 7_e, were over 0.8 in the model with SG2 + scaling (Fig. 2A). The prediction models for most of the clusters in Clus 2 did not work well, perhaps because of inconsistent fluctuation in the expression (Takehisa *et al.* 2022). Furthermore, we tried to test the prediction model using datasets for all the points under andosol conditions, and at 28, 42, 56, and 70 DAT under alluvial conditions, as training data and predicted the expression at five points, i.e., 21, 35, 49, 63, and 77 DAT, under alluvial conditions. In many clusters, the predicted values were similar to the expression levels, and differences between *japonica* and *indica* cultivars were reproduced in several clusters, such as Clus 10_a and Clus 7_e (Fig. 2B, Supplemental Fig. 2).

### Hyperspectral imaging of rice leaves throughout the growth process

**Fig. 3.**
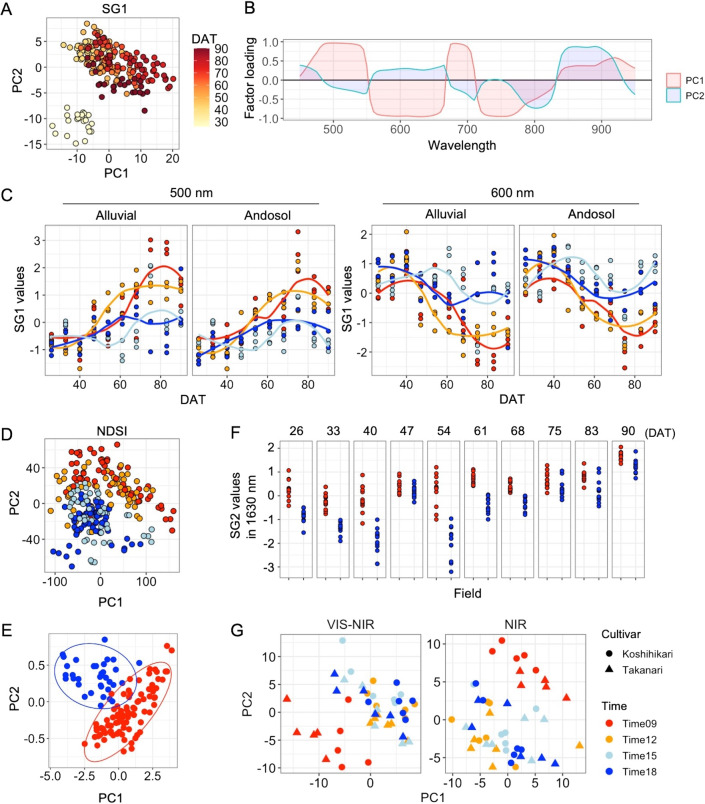
Hyperspectral imaging for clarification of genetic and physiological signatures. (A) PCA using SG1 pretreated values of VIS-NIR. Plots are colored based on DAT. (B) Factor loading for PC1 (red) and PC2 (blue). (C) Profiles of SG1 pretreated values at 500 nm and 600 nm during the growth process. Red, orange, blue, and light blue plots and lines show Nipponbare, Koshihikari, Takanari, and IR64, respectively. (D) PCA using NDSI of VIS-NIR. Red, orange, blue, and lightblue plots show Nipponbare, Koshihikari, Takanari, and IR64, respectively. (E) PCA using NDSI of VIS-NIR hyperspectral imaging data in flag leaves of 10 *japonica* and four *indica* cultivars at the heading stage under alluvial soil field conditions. Red and blue plots show *japonica* and *indica* cultivars, respectively. (F) Profiles of SG2 pretreated values at 1630 nm during the growth process. Red and blue plots represent alluvial and andosol soil conditions, respectively. (G) PCA using SG2 pretreated values of VIS-NIR and NIR for Koshihikari and Takanari at four time points during the day at 35 DAT.

We further carried out hyperspectral imaging of the 4 rice cultivars at 26, 33, 40, 47, 54, 61, 68, 75, 83, and 90 DATs under alluvial and andosol soil field conditions in 2020 by using the 2 systems for VIS-NIR (450–950 nm) and NIR (950–1650 nm) (Table 1). The reflectance spectrum was pretreated with smoothing, SG1, and SG2, and then subjected to PCA. We also used NDSI for the analysis (Supplemental Fig. 3A, 3B). In accordance with the distribution of each sample revealed by PCA, we first focused on the PC scores of SG1 in VIS-NIR, which seemed to reflect the growth-processes well; PC1 was associated with growth stages and PC2 mainly detected differences between samples at 26 DAT and the others (Fig. 3A). Factor loading analyses clarified that SG1 values of 485–540 nm and 675–695 nm and those of 580–655 nm and 725–765 nm were positively and negatively correlated with PC1 scores, respectively (Fig. 3B). Indeed, the profiles of SG1 + scaling values at 500 and 600 nm tended to be up and down along the growth direction, respectively, and detected differences between subspecies at the late stages (Fig. 3C). Although PC2 scores were positively associated with SG1 values at 840–890 nm, further analysis with sufficient samples at earlier stages of the growth is needed to clarify the relationship between the wavelength and biological phenomena. Next, we focused on the PC scores for NDSI in VIS-NIR, which seemed to be able to divide the samples into subspecies more clearly (Fig. 3D, Supplemental Fig. 3A). In order to confirm their potential, we performed PCA using smoothing, SG1, SG2, and NDSI for each DAT (Supplemental Fig. 4). Overall, NDSI enabled us to distinguish *japonica* and *indica* cultivars throughout growth with PC1 and PC2 scores more clearly than the other pretreated data, regardless of field soil conditions (Supplemental Table 1). Furthermore, we analyzed VIS-NIR hyperspectral imaging data for flag leaves in 10 *japonica* and four *indica* cultivars at the heading stage under the alluvial soil field conditions. PCA with NDSI values showed that VIS-NIR data had the potential to distinguish the subspecies, regardless of differences in the number of days to heading (Fig. 3E).

As for differences between field conditions, PC3 and PC4 scores derived from SG2 in NIR were able to distinguish alluvial soil from andosol throughout the entire growth process, whereas PC1 and PC2 scores could not (Supplemental Fig. 5A, 5B, Supplemental Table 1). Factor loading analysis at each DAT revealed that the 1600–1650 nm spectral region was associated with the PC3 scores at 26, 33, 40, 47, 54, 61, and 68 DAT, and with the PC4 scores at 83 and 90 DAT, which mainly reflected differences between the two soil conditions (Supplemental Fig. 5B, 5C). SG2 values at 1630 nm tended to be higher for alluvial soil than for andosol, especially at the early stage of growth (Fig. 3F). Although phosphate retention capacity is one of the biggest differences between the two soil conditions and rice plants promote the expression of key genes associated with P acquisition and recycling in andosol soil only during the tillering stage, the overall signatures of rice plants throughout their entire growth were similar between the soil conditions, in terms of gene expression level (Takehisa *et al.* 2022). Therefore, we further analyzed the NIR hyperspectral imaging data for flag leaves derived from eight *japonica* cultivars at the heading stage under the two conditions. This revealed no distinction between the conditions in PCA with SG2, but SG2 scores for andosol soil at1630 nm were lower only for cultivars with early heading (Supplemental Fig. 6).

As a large number of leaf transcripts are known to exhibit a diurnal cycling pattern (Hayes *et al.* 2010, Nagano *et al.* 2012), we further collected hyperspectral imaging data for leaves of ‘Koshihikari’ and ‘Takanari’ at four time points, i.e., 9:00, 12:00, 15:00, and 18:00 at 35 DAT. PCA with SG2 distinguished the data at 9:00 from the others in both VIS-NIR and NIR (Fig. 3G).

### Reflectance spectroscopy analysis of rice grains

**Fig. 4.**
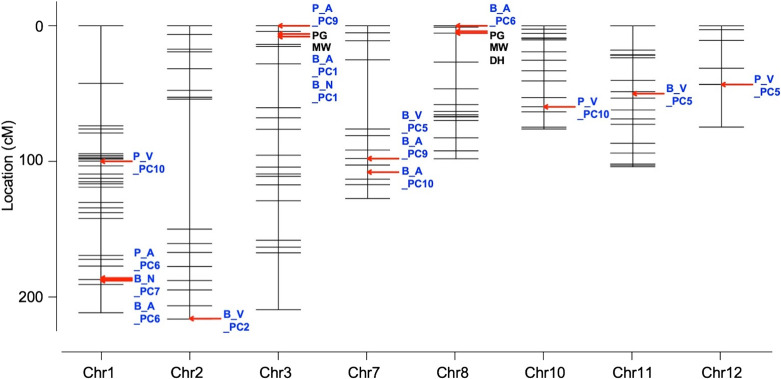
Positions of the detected QTLs. DH: days to heading, PG: perfect grain, MW: milky white grain. Details are shown in Supplemental Table 2.

Reflectance spectroscopy has been widely applied for evaluation of the nutritional qualities of various agricultural products, such as protein and sugar contents. In the present study, we obtained the hyperspectral profiles of brown rice grains and their ground powder for 96 RILs derived from ‘Emi-no-kizuna’ and ‘Tomohonami’. ‘Emi-no-kizuna’ has strong resistance to high temperature at the ripening stage and the brown rice grains have a favorable appearance (Nagaoka *et al.* 2017). The hyperspectral profiles we obtained were subjected to SG2 + scaling and then divided into two parts, i.e., VIS: 450–750 nm and NIR: 750–2499.5 nm. We then obtained the scores of principal components (PC) 1–10 in terms of ALL (450–2499.5 nm), VIS and NIR, respectively. Supplemental Fig. 7 shows the distribution of the PC scores for ‘Emi-no-kizuna’, ‘Tomohonami’, and the 96 RILs. The five measured traits, i.e., DH, PG, MW, BW, and WB, and each the PC scores were subjected to QTL analysis (Fig. 4, Supplemental Table 2). The numbers of QTLs associated with PC scores derived from the ground powder and brown rice grains were five and 10, respectively. QTLs associated with PG and MW were detected in two regions, i.e., chromosome (Chr) 3 and Chr 8, while those associated with BW and WB were not. Two of the QTLs related to grain appearance (GA) traits were co-localized or located close to some of the QTLs for PC scores. In regions close to the QTL on Chr 3, QTLs for PC9 of ground powder with ALL (P_A_PC9), PC1 of brown rice grain with ALL (B_A_PC1), and PC1 of brown rice grain with NIR (B_N_PC1) were also detected. On Chr 8, in addition to the QTLs for PG and MW, QTLs for DH and B_A_PC6 were detected. Nagaoka *et al.* (2019) identified QTLs associated with GA traits in seven regions including the two regions where QTLs for PG and MW were identified in the present study. In the Chr 7 region where the QTL of WB has been reported (Nagaoka *et al.* 2019), QTLs related to PC5 for brown rice grain with VIS (B_V_PC5), B_A_PC9, and B_A_PC10 were identified. We were unable to detect any QTLs on Chr 4, even though the QTL with the largest phenotypic variation in PG has been reported on Chr 4 (Nagaoka *et al.* 2019). Interestingly, QTLs related to three PC scores, i.e., P_A_PC6, B_N_PC7, and B_A_PC6, were identified at the end of the long arm of Chr 1, where *sd1* is located. It has been reported that PG was associated with the genotype of *sd1* but no QTLs controlling GA traits were detected at the region around *sd1* locus (Nagaoka *et al.* 2019).

## Discussion

### Hyperspectral profiles of rice leaves

Rice plants undergo a drastic change in N and P nutrient status around 30–40 DAT (Takehisa and Sato 2019). Thus, there would be notable differences in physiological statuses between the early and late stages of growth. Therefore, it is considered that hyperspectral profiling of leaves enabled us to detect growth-stage dependent features in both reflectance spectroscopy and hyperspectral imaging (Figs. 1A, 3A, Supplemental Fig. 3), and that the prediction of the expression of clusters with up- and down-regulation tended to work well (Fig. 2, Supplemental Fig. 2). The prediction models for eight clusters including Clus 10_a, Clus 10_c, and Clus 8_d showed good performance (Fig. 2). Clus 10_a and 10_c, showing a downward trend, contain cytokinin-metabolism related genes such as the adenosine phosphate-isopentenyltransferase genes (*OsIPT4* and *OsIPT7*) (Sakamoto *et al.* 2006) and the cytokinin dehydrogenase gene (*OsCKX3*) (Ashikari *et al.* 2005). Cytokinin plays an important role in the regulation of tiller bud outgrowth together with nitrogen (Ohashi *et al.* 2017, Xu *et al.* 2015). On the other hand, *OsMADS14*, which is associated with reproductive phase transition (Kobayashi *et al.* 2012), is included in Clus 8_d, which showed an upward trend. These results suggested that reflectance spectroscopy has the potential to detect the transition of physiological status associated with morphological determination, and further might also be a potential labor- and cost-effective alternative for analyzing the expression profiles of such developmentally regulated genes.

In the growth of rice plants, N status becomes deficient followed by suppression of tiller bud elongation, and expression of genes for acquisition and recycling of phosphorus (P) is observed only under andosol soil conditions with low available P during the tillering stage (Takehisa and Sato 2019). Therefore, there are differences in rice plants especially at early stage of growth between alluvial and andosol soil conditions. In this study, we detected field-condition dependent differences not only at 28, 35, and 42 DATs with reflectance spectroscopy but also throughout entire growth with hyperspectral imaging (Fig. 1B, Supplemental Fig. 5). Although it was unclear whether latter represents a common difference between alluvial and andosol soil conditions, or was a feature specific to the fields used in the present study, it was not detected in our previous comprehensive transcriptome analysis (Takehisa *et al.* 2022).

In the prediction of gene expression with reflectance spectroscopy, the differences between *japonica* and *indica* cultivars were reproduced in several clusters, such as Clus 10_a and Clus 7_e (Fig. 2B). In addition, hyperspectral imaging enabled us to detect subspecies-specific features of flag leaves at the heading stage (Fig. 3E). Although it has been unclear whether differences between subspecies reflect specific physiological parameters based on genetic background, the high-yielding *indica* cultivar, ‘Takanari’, has been reported to have a high potential in terms of leaf photosynthesis rate (Takai *et al.* 2013). Therefore, further study to clarify the relationship between the hyperspectral profile and this feature might aid more effective evaluation of source capacity.

Hyperspectral imaging techniques are based on the interaction between light and plant tissue, and the spectrum of sunlight or artificial light varies according to light quality, light source, and light angle. In this study, we captured hyperspectral images of detached leaves under laboratory conditions, thereby yielding a series of data acquired under uniform conditions. In addition, we used smoothing, SG1, and SG2 pretreatment methods and NDSI for analysis of hyperspectral imaging data, and focused PC scores derived from SG1, SG2, and NDSI to detect features for growth stages, field conditions, and subspecies, respectively. It has been shown that normalization with NDSI reduces the error factor attributable to observation conditions and background effects and allows the relationship of samples to be linearized (Inoue *et al.* 2008). In addition, we showed that profiles of the sample at 9:00 were distinguished from those at 12:00, 15:00, and 18:00 (Fig. 3G), implying that the sampling time point should be taken into consideration when attempting leaf hyperspectral image capture. Therefore, our results suggest that hyperspectral imaging is useful for understanding overall signatures in both genetic and physiological parameters with high resolution, at least in the cases where the data are uniformly captured and appropriately pretreated. On the other hand, our results also demonstrated that leaf hyperspectral profiles were changed depending on growth stages, times, field conditions and genetic backgrounds. This means that it is important to prepare hyperspectral profiles with sufficient varieties for development of a robust prediction model under field conditions.

### Hyperspectral profiles of rice grains

Multiple QTLs, including of those for the PC scores, were identified in the regions where QTLs were associated with GA, and almost all of the QTLs of the PC scores were derived from ALL and NIR (Fig. 4, Supplemental Table 2), suggesting that near-infrared spectroscopy may be needed for evaluation of GA traits such as PG and MW. On the other hand, the other QTLs for PC scores, i.e., P_V_PC10 on Chr 1 and Chr 10, B_V_PC2 on Chr 2, B_V_PC5 on Chr 11, and P_V_PC5 on Chr 12, were derived from VIS and showed relatively low LOD values. This suggests that these QTLs control the hue of the grain surface, or have a minor effect on the grain as a whole. High-quality hyperspectral profiles are thought to contain various types of information related to physiological and genetic traits. However, it would not be an easy task to extract all biological parameters fully using this technique alone. GA is known to be a complex trait controlled by not only genetic but also environmental factors. Accordingly, clarification of the relationship between the identified PC scores and GA traits using a combination of the VIS-NIR analysis and the genetic studies could possibly lead to the development of a new indicator for evaluation of GA traits.

## Author Contribution Statement

Y.S. and H.T. designed the research. H.T., Y.S. and A.I. analyzed the spectral data using reflectance spectroscopy. H.T. and Y.S. analyzed the spectral data using the spectral imaging system. Y.S. and I.N. performed the QTL analysis. Y.S. and H.T. wrote the paper.

## Supplementary Material

Supplemental Figures

Supplemental Tables

## Figures and Tables

**Table 1.** Summary of hyperspectral data used in the study

Experiments	Instruments	Spectral range*^a^*	Sample information							Spectral pretreatment methods*^b^*	Analytical methods	Biological insights
			Years	Fields	Number of cultivars/lines	Growth stages	Samples	Sample form	Number of samples			
Reflectance spectroscopy analysis of rice leaf throughout growing process	Spectrometer	400–2499.5 nm with 4200 bands	2016	Alluvial Andosol	*japonica*: 2 *indica*: 2	9 points (21–77 DAT)	Leaf	Grinded powder	72	SG2 + scaling	PCA PLS-R	Growth dynamics Prediction of gene expression
Hyperspectral imaging of rice leaf throughout growing process	VIS-NIR hyperspectral camera	450–950 nm with 101 bands	2020	Alluvial Andosol	*japonica*: 2 *indica*: 2	10 points (26–90 DAT)	Leaf	Detached	240	SG1 + scaling NDSI	PCA	Growth dynamics Difference between subspecies
			2020	Alluvial	*japonica*: 10 *indica*: 4	Heading	Flag leaf	Detached	126	NDSI	PCA	Difference between subspecies
			2020	Alluvial	*japonica*: 1 *indica*: 1	35 DAT (9:00, 12:00, 15:00, 18:00)	Leaf	Detached	46	SG2 + scaling	PCA	Diurnal cycle pattern
	NIR hyperspectral camera	950–1650 nm with 71 bands	2020	Alluvial Andosol	*japonica*: 2 *indica*: 2	10 points (26–90 DAT)	Leaf	Detached	240	SG2 + scaling	PCA	Difference between field conditions
			2020	Alluvial Andosol	*japonica*: 8	Heading	Flag leaf	Detached	120	SG2 + scaling	PCA	Difference between field conditions
			2020	Alluvial	*japonica*: 1 *indica*: 1	35 DAT (9:00, 12:00, 15:00, 18:00)	Leaf	Detached	46	SG2 + scaling	PCA	Diurnal cycle pattern
Reflectance spectroscopy analysis of rice grain	Spectrometer	400–2499.5 nm with 4200 bands	2020	Alluvial	96 RILs	after harvesting	Brown rice grain	Intact	96	SG2 + scaling	PCA QTL	Grain appearance
			2020	Alluvial	96 RILs	after harvesting	Brown rice grain	Grinded powder	96	SG2 + scaling	PCA QTL	Grain appearance

*^a^* Spectral range used for the analysis. *^b^* Methods used in the main analysis.
