# Supplementary material for: Visible-near infrared spectral analysis for identification of physiological and genetic features in rice
Source: Breed Sci. 2025 Oct 7;75(5):349–57. doi: 10.1270/jsbbs.25018 (PMC13129574; doi:10.1270/jsbbs.25018)
Supplement: Supplementary file 1 — Supplemental Figures [file 75_349_s1.pdf]

doi: 10.1270/jsbbs.25018

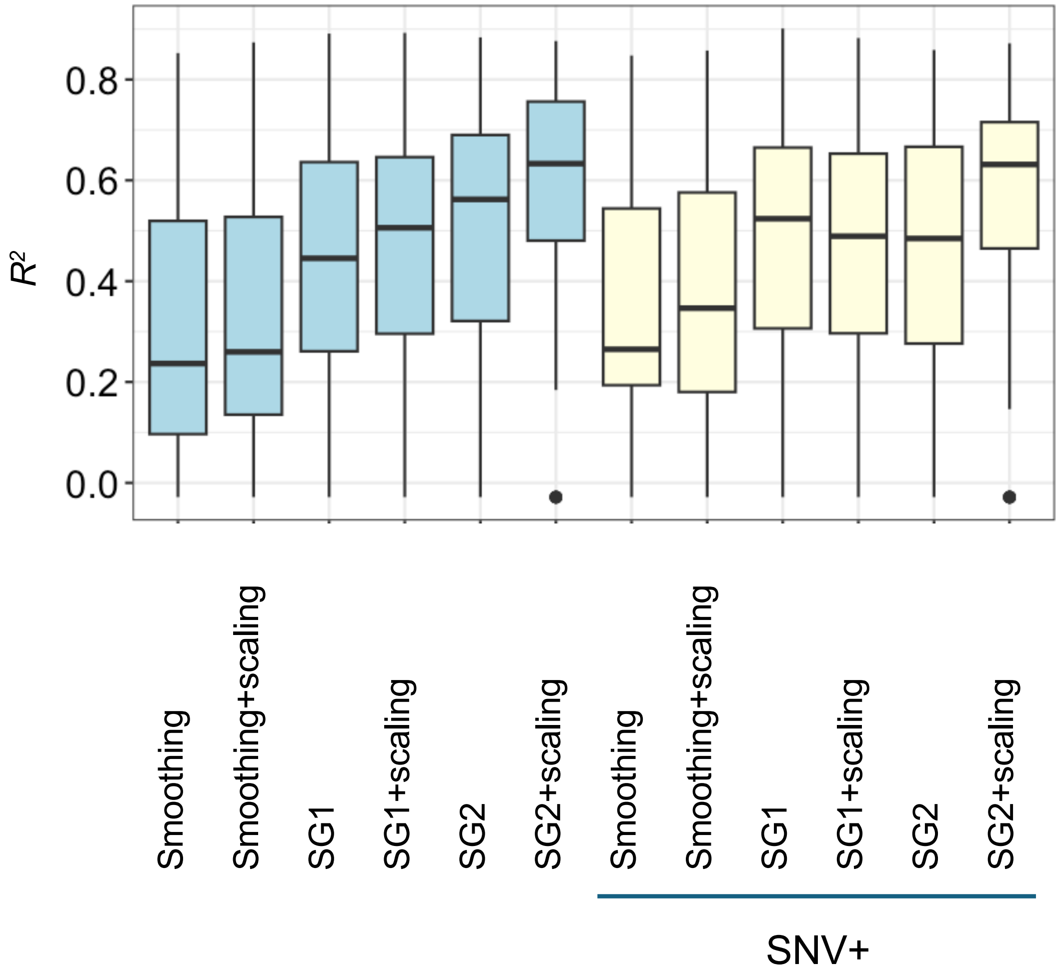

Supplemental Fig. 1.
Evaluation of the prediction models with 12 sets of pretreated data. Coefficient of determination (R^2) after leave-one-out cross validation in the prediction model relative to averaged expression values in the 54 clusters was plotted for each pretreatment method.

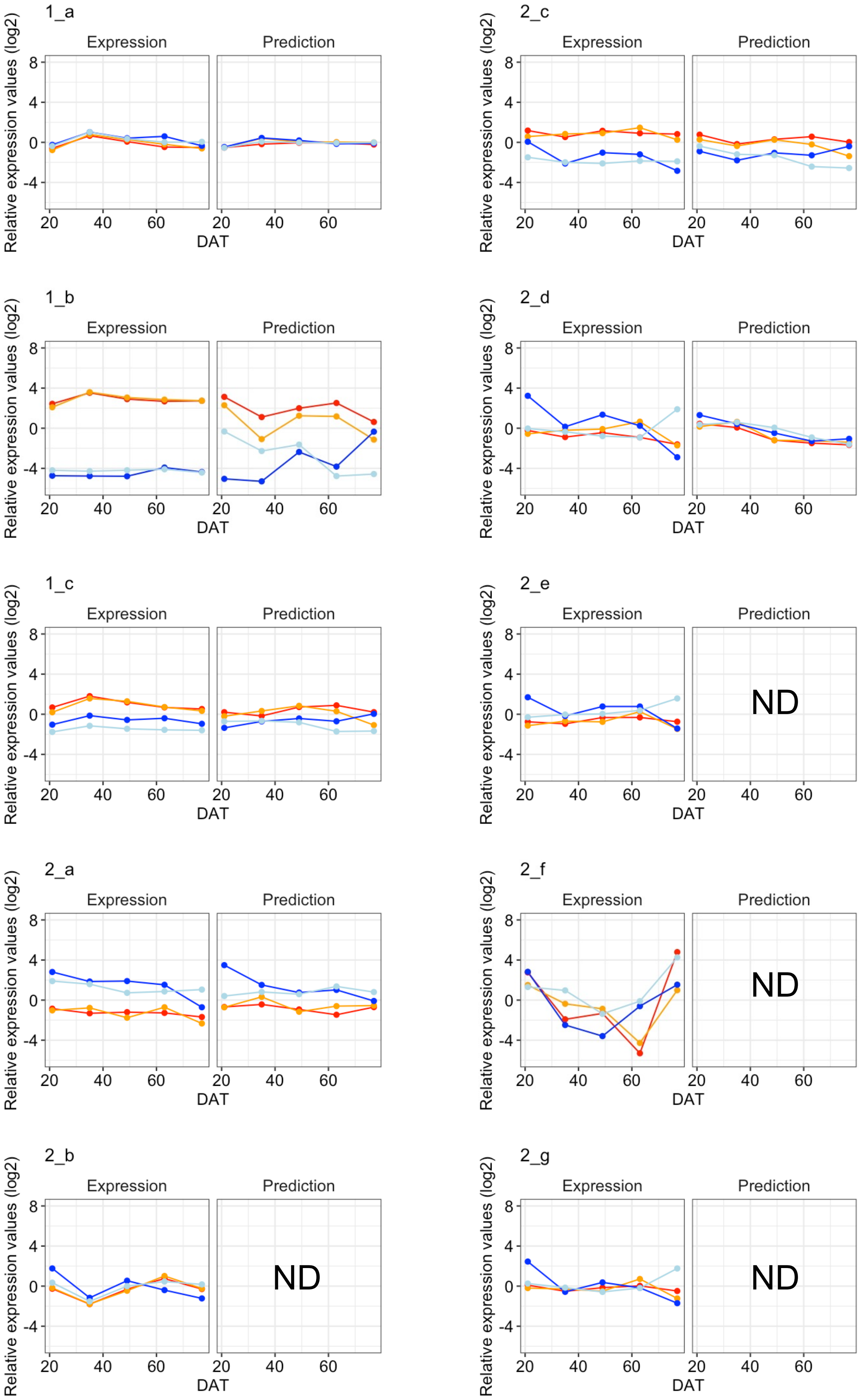

Supplemental Fig. 2 (Continued)

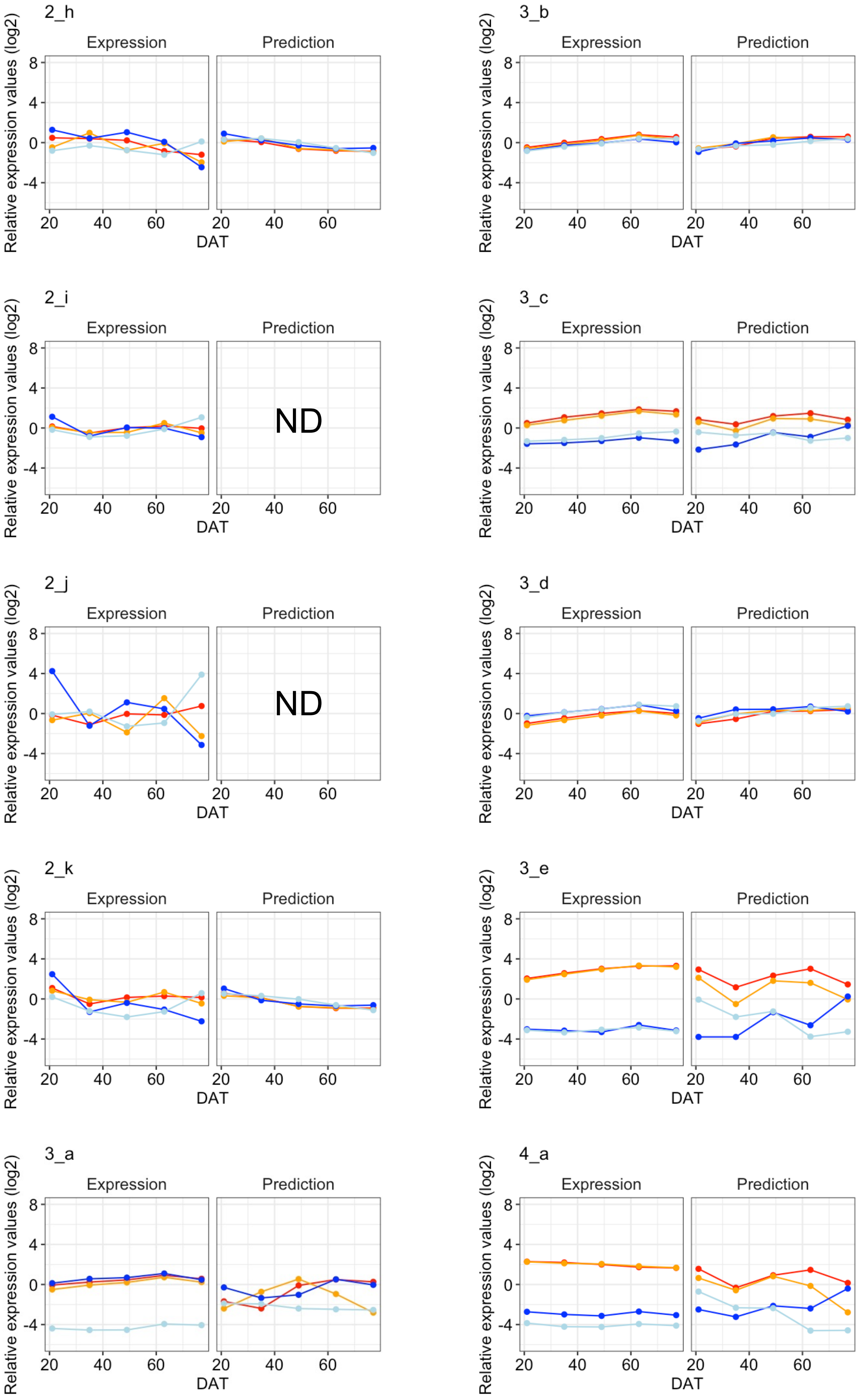

Supplemental Fig. 2 (Continued)

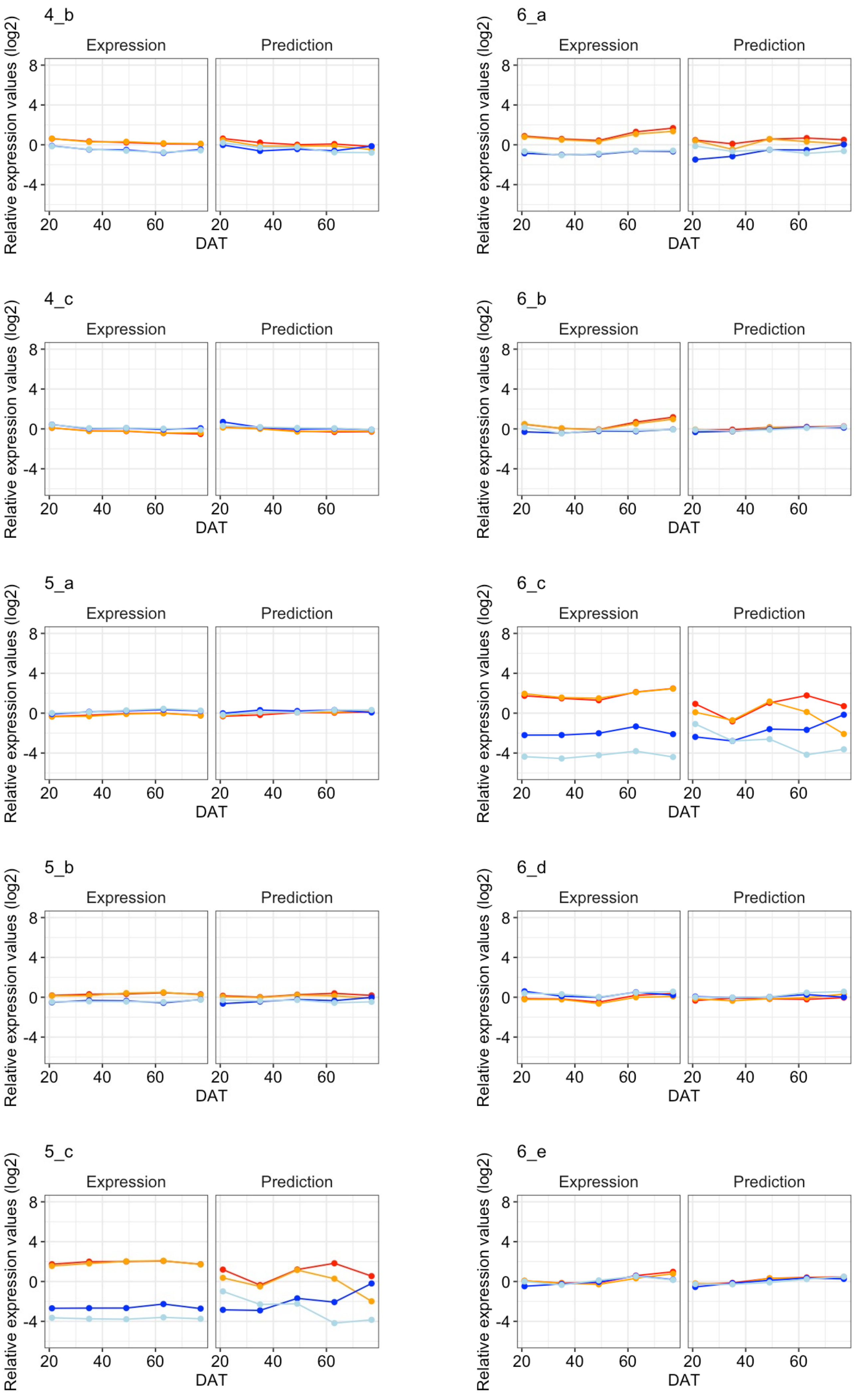

Supplemental Fig. 2 (Continued)

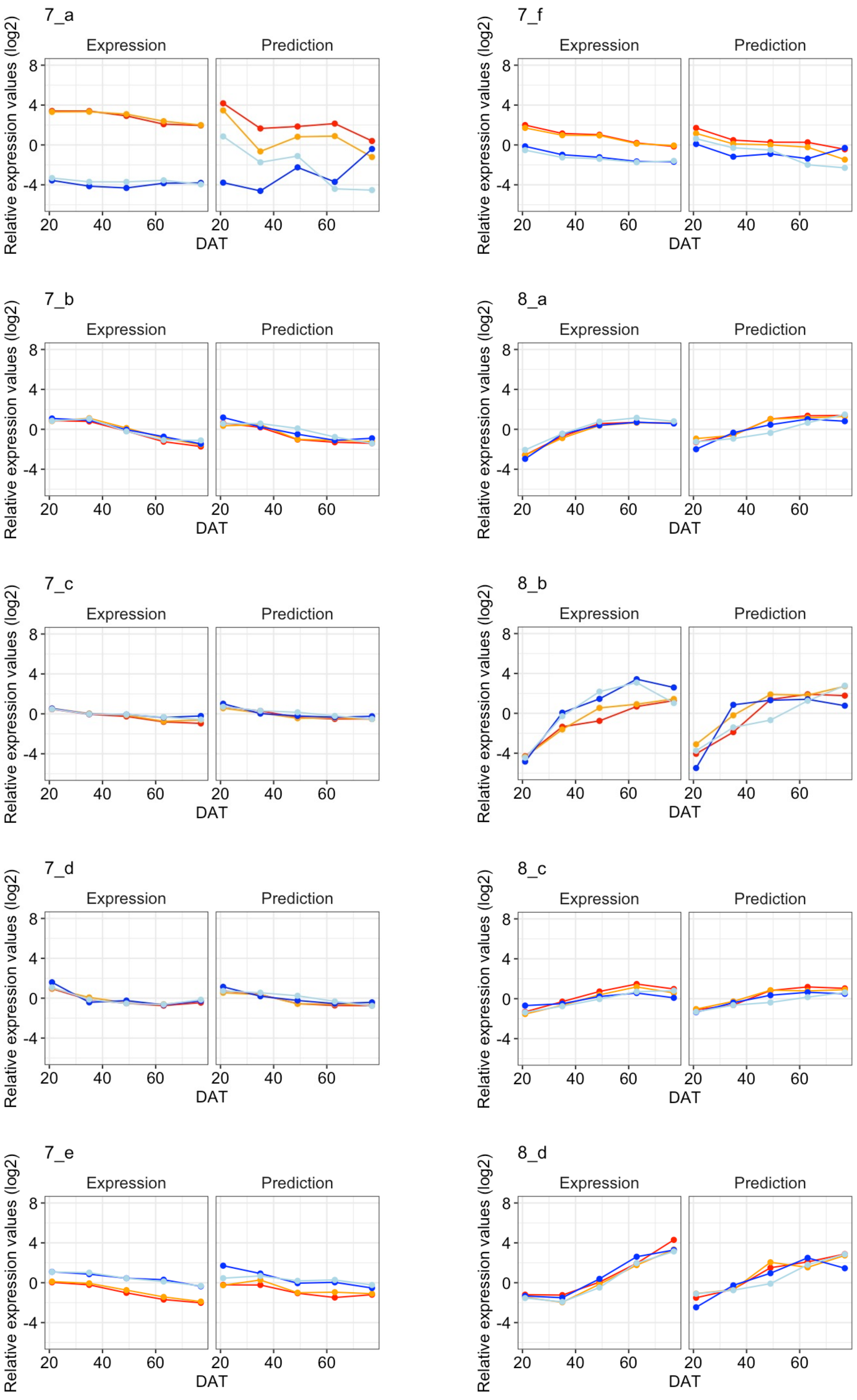

Supplemental Fig. 2 (Continued)

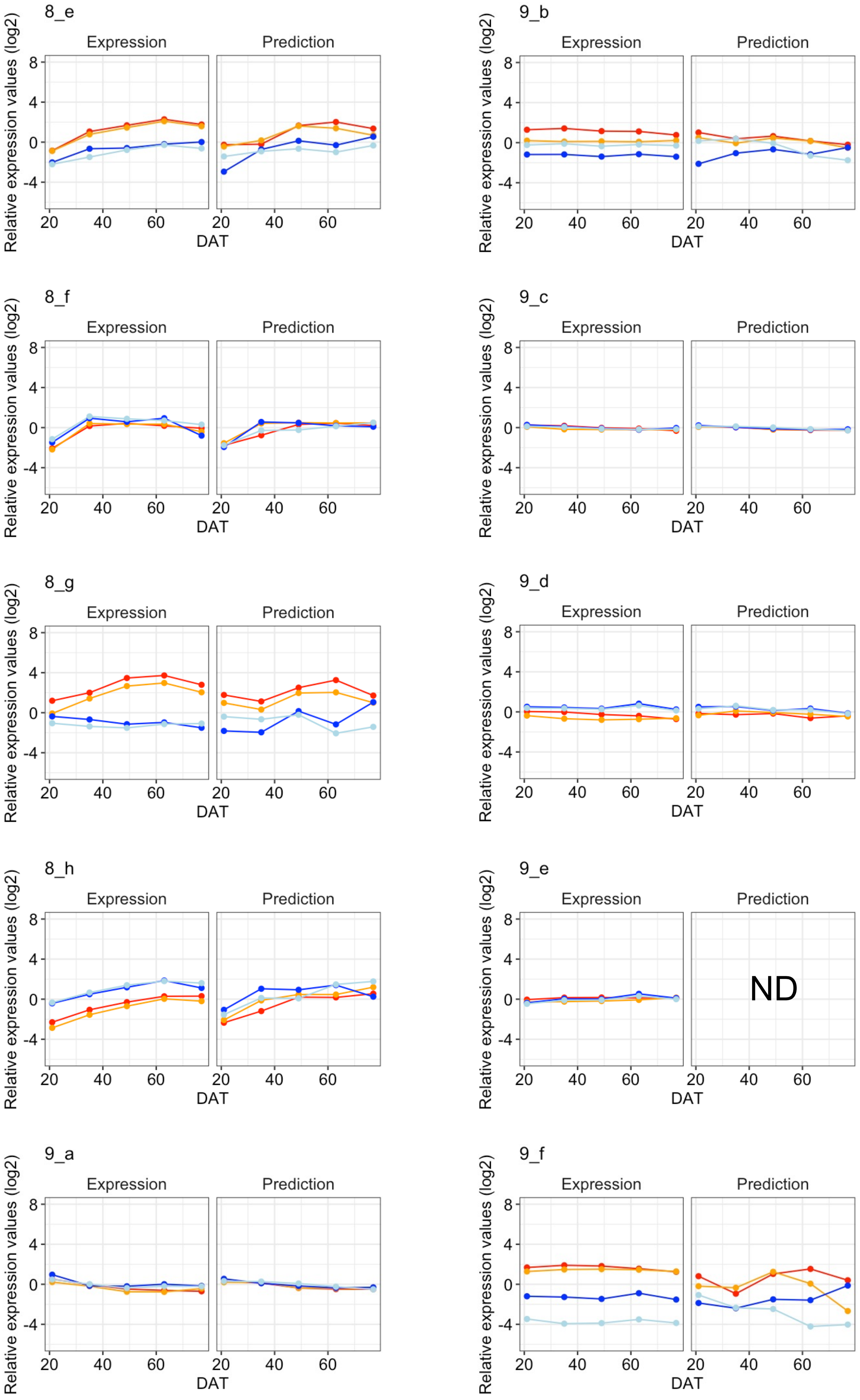

Supplemental Fig. 2 (Continued)

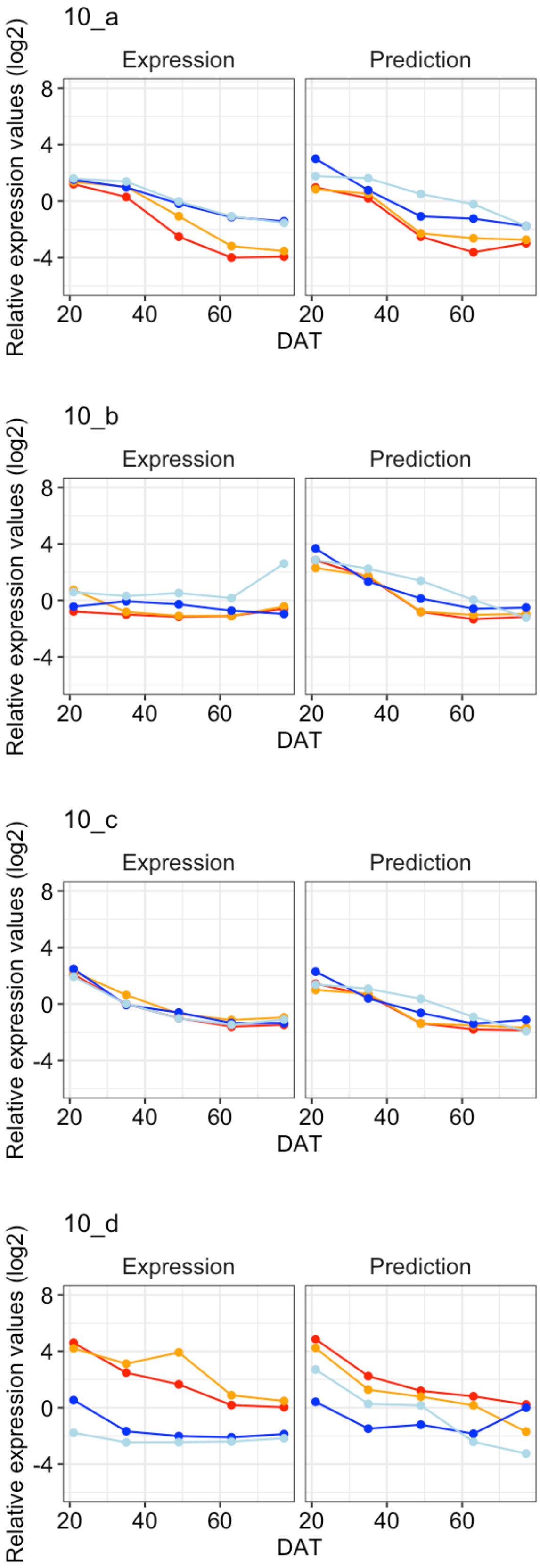

Supplemental Fig. 2.
Comparison between expression and predicted values in the 54 clusters. Red, orange, blue, and light blue lines represent Nipponbare, Koshihikari, Takanari, and IR64, respectively.

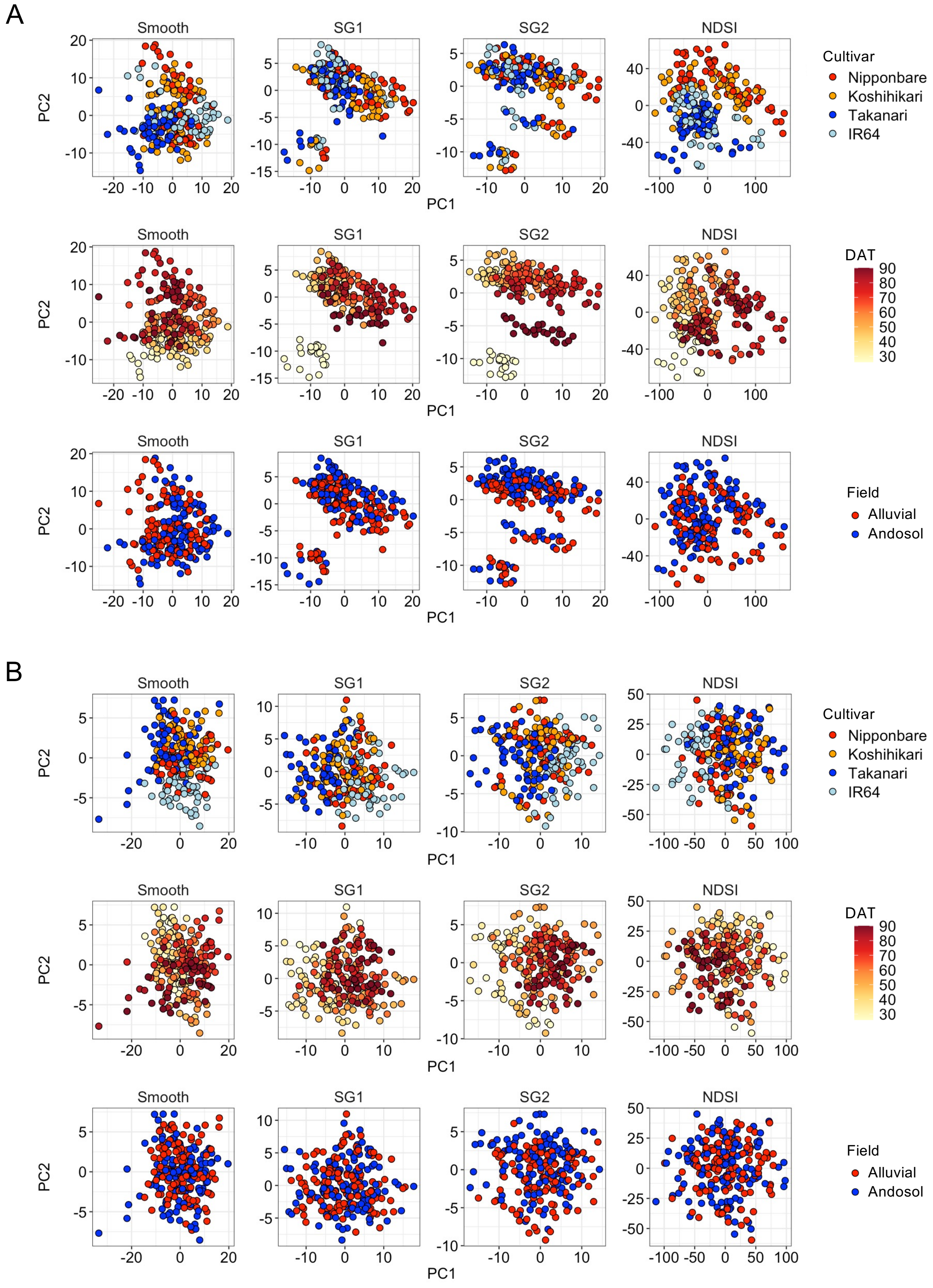

Supplemental Fig. 3.

PCA of hyperspectral imaging data with VIS-NIR (A) and NIR (B) from the four rice cultivars at 10 time points from 26 to 90 DAT under the two field conditions. Pretreated data with smoothing, SG1, and SG2 and NDSI were used for this analysis. Colors of plots indicate cultivars (upper), DATs (middle), and fields (lower), respectively.

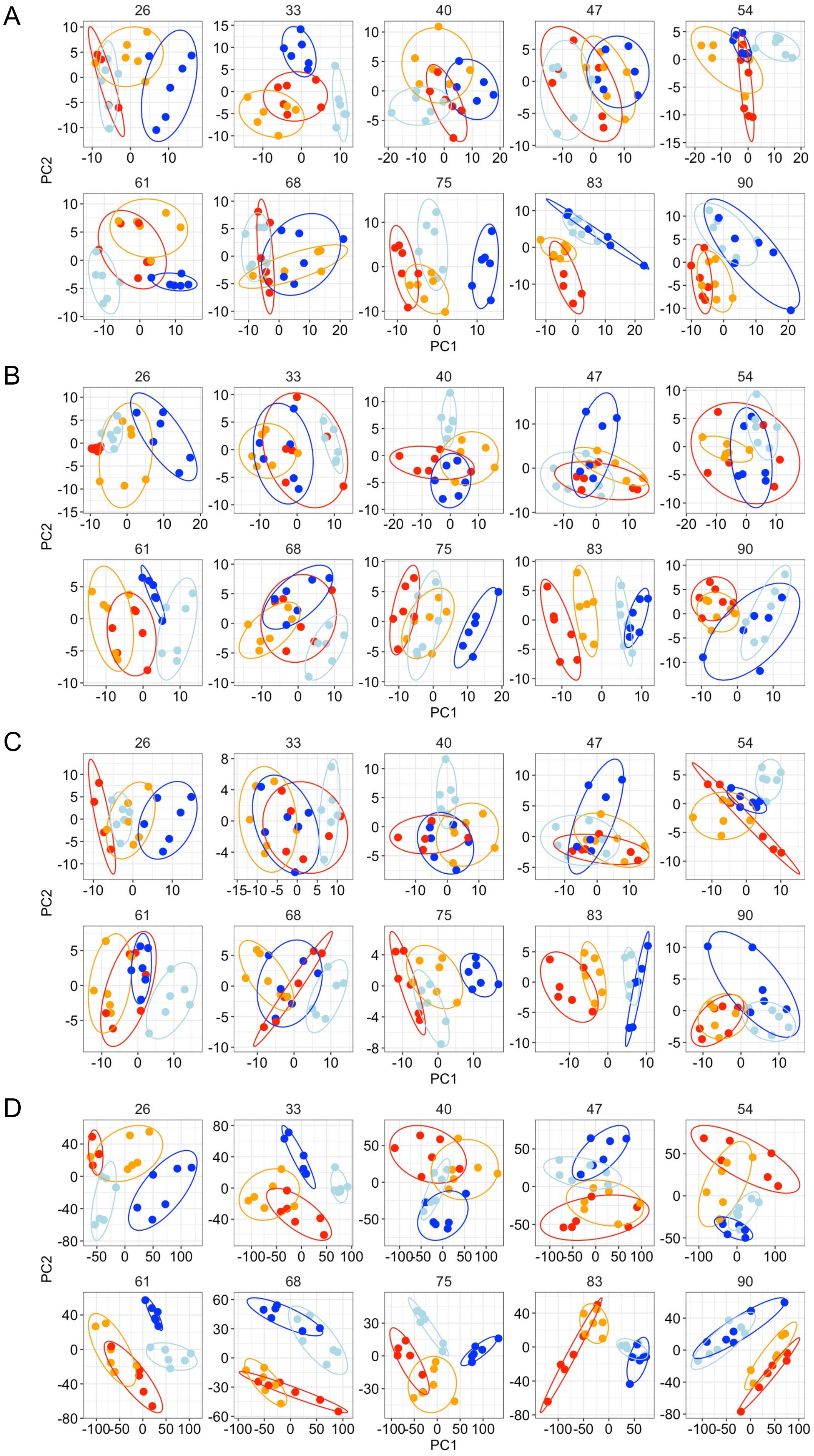

Supplemental Fig. 4.

PCA of VIS-NIR hyperspectral imaging data with smoothing (A), SG1 (B), SG2 (C), and NDSI (D) from the four rice cultivars under the two field conditions at each DAT. Red, orange, blue, and light blue plots represent Nipponbare, Koshihikari, Takanari, and IR64, respectively.

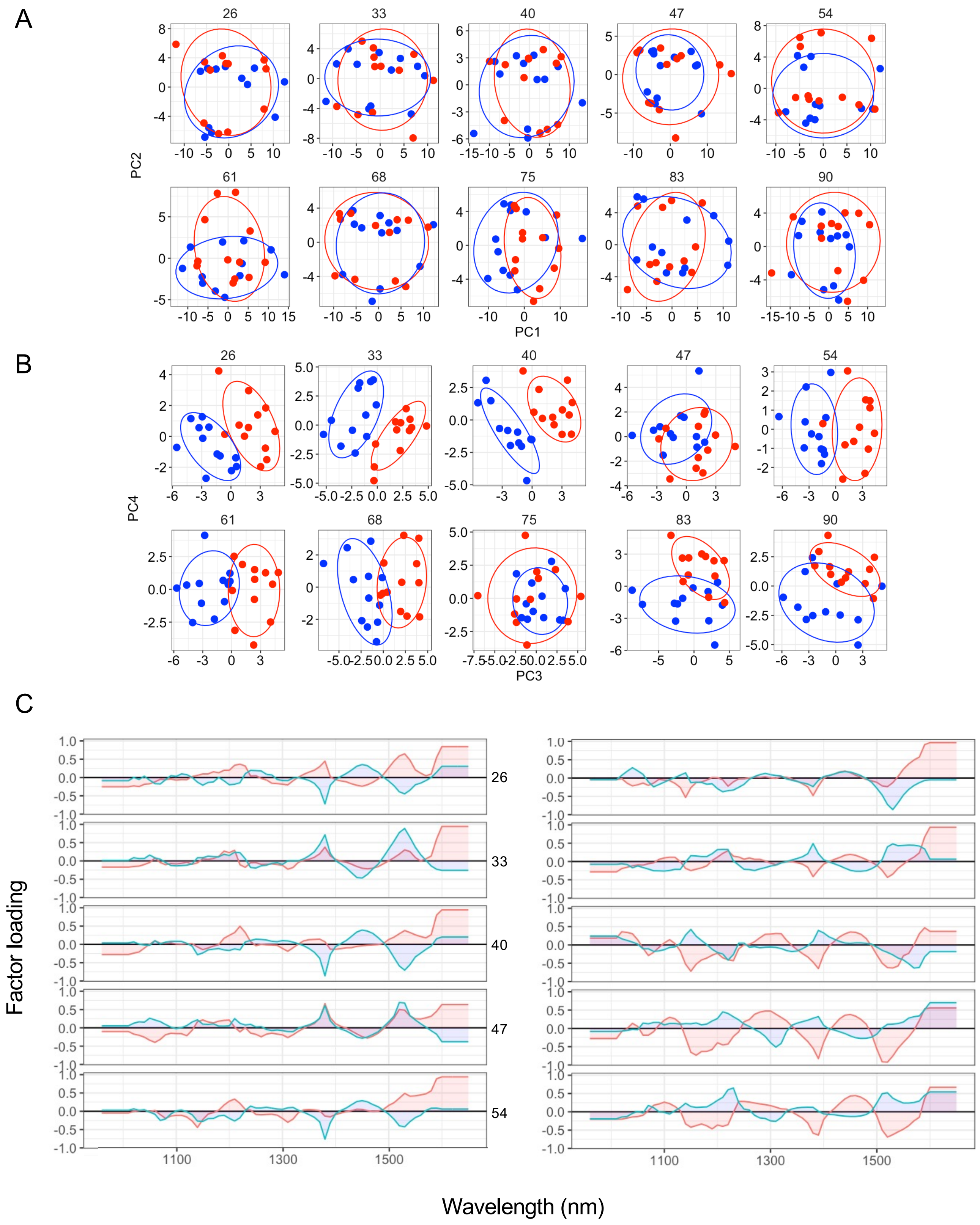

Supplemental Fig. 5.
PCA of NIR hyperspectral imaging data with SG2 pretreated values derived from the four rice cultivars under the two field conditions at each DAT. Plot graphs for PC1 and PC2 (A) and for PC3 and PC4 (B). Red and blue plots represent alluvial and andosol soil conditions, respectively. (C) Factor loadings for PC3 (red) and PC4 (blue).

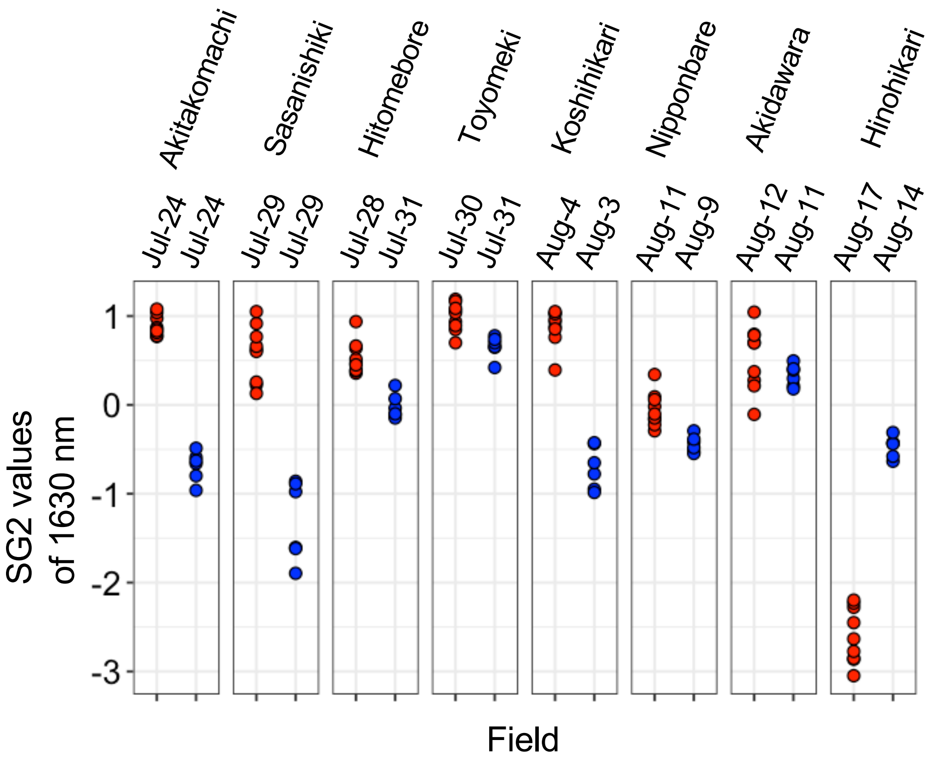

Supplemental Fig. 6.
Profile of SG2 pretreated values of NIR at 1630 nm for eight *japonica* cultivars at the heading stage. Red and blue plots show alluvial and andosol soil conditions, respectively. Dates indicate the time points when hyperspectral imaging data were obtained.

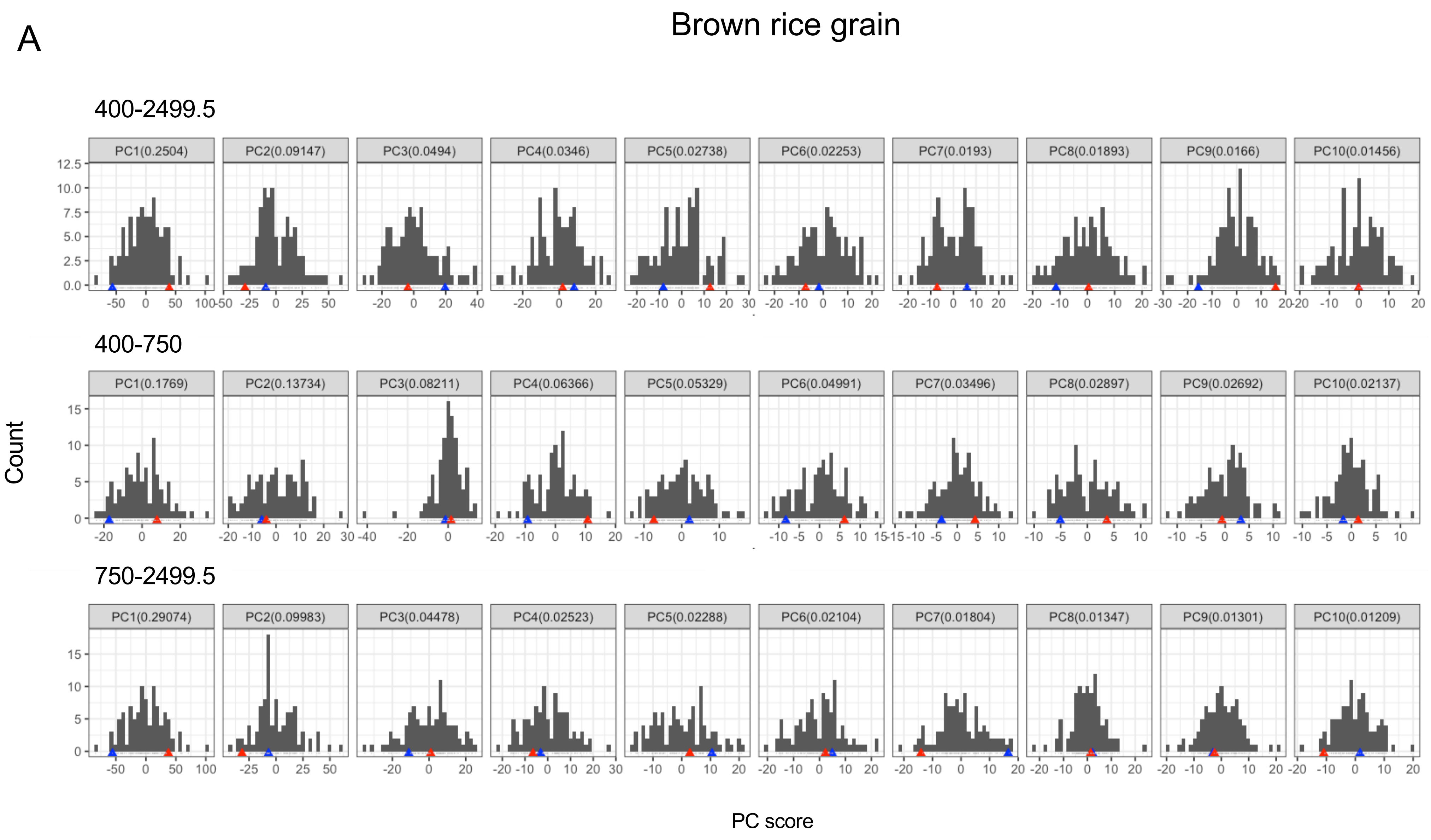

Supplemental Fig. 7 (Continued)

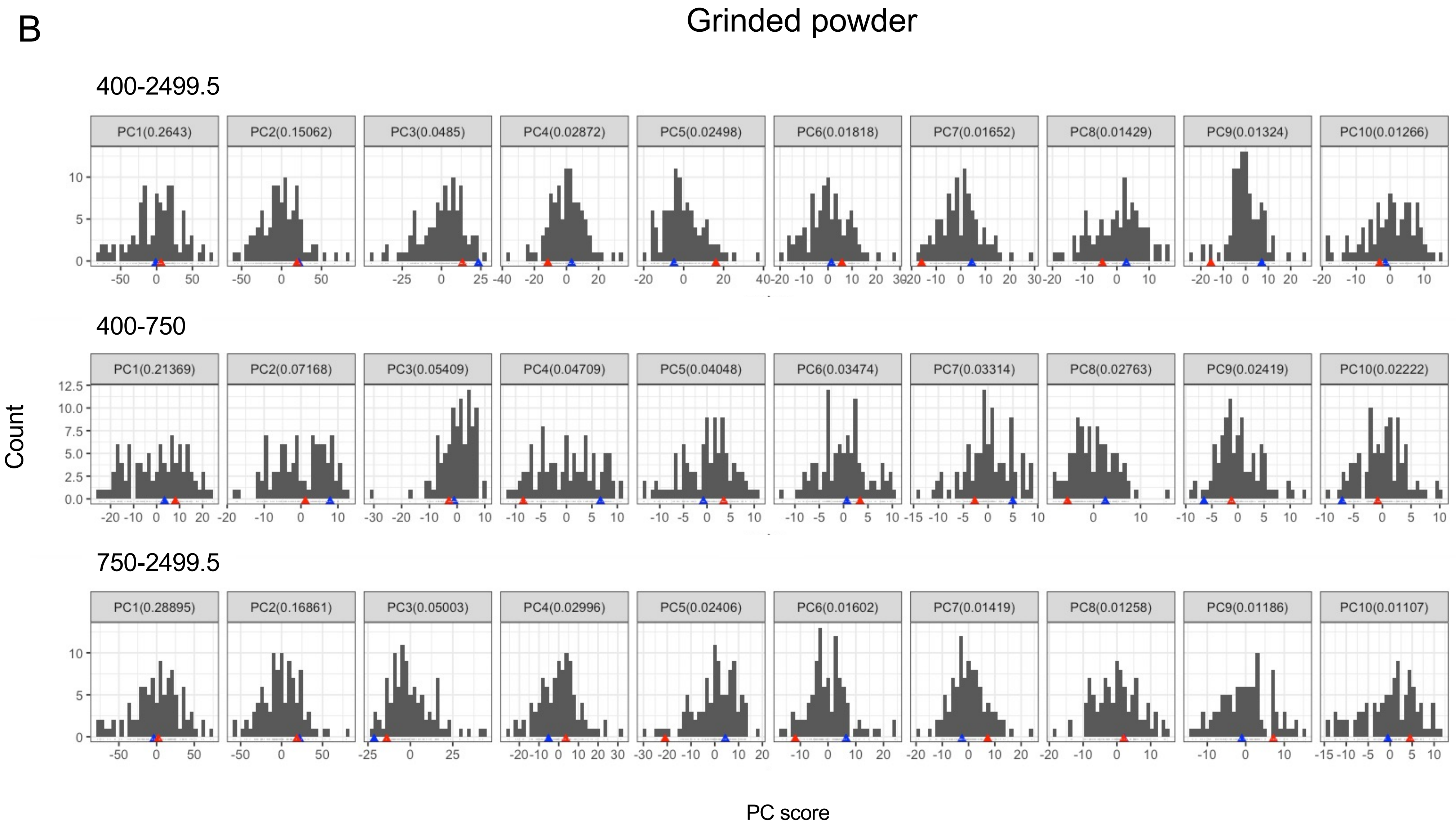

Supplemental Fig. 7.
Distribution of PC1-10 scores for Emi-no-kizuna and Tomohonami with brown rice grains (A) and ground powder (B).
Arrowheads under graphs indicate the positions of Emi-no-Kizuna (red) and Tomohonami (blue).
