# Supplementary material for: Visible-near infrared spectral analysis for identification of physiological and genetic features in rice
Source: Breed Sci. 2025 Oct 7;75(5):349–57. doi: 10.1270/jsbbs.25018 (PMC13129574; doi:10.1270/jsbbs.25018)
Supplement: Supplementary file 2 — Supplemental Tables [file 75_349_s2.pdf]

doi: 10.1270/jsbbs.25018

Supplemental Table 1. Classification accuracy with discriminant analysis using PC scores

Targets	PC scores	Pretreatment methods	Accuracy										
			26 DAT	33 DAT	40 DAT	47 DAT	54 DAT	61 DAT	68 DAT	75 DAT	83 DAT	90 DAT	Average
Subspecies	PC1, PC2	Smoothing	0.68	0.92	0.67	0.58	0.92	0.79	0.58	0.92	1.00	1.00	0.81
		SG1	0.68	0.63	0.54	0.58	0.75	1.00	0.75	0.71	1.00	0.96	0.76
		SG2	0.55	0.71	0.67	0.67	0.96	0.88	0.75	0.75	1.00	0.96	0.79
		NDSI	0.91	0.96	0.79	0.96	0.92	1.00	1.00	1.00	1.00	1.00	0.95
Field	PC1, PC2	Smoothing	0.67	0.63	0.63	0.71	0.63	0.58	0.46	0.75	0.58	0.54	0.62
		SG1	0.54	0.58	0.50	0.50	0.58	0.63	0.50	0.67	0.54	0.50	0.55
		SG2	0.58	0.42	0.50	0.54	0.50	0.54	0.46	0.63	0.46	0.54	0.52
		NDSI	0.54	0.58	0.50	0.58	0.58	0.54	0.54	0.71	0.54	0.50	0.56
Field	PC3, PC4	Smoothing	0.63	0.54	0.75	0.54	0.83	0.75	0.54	0.58	0.63	0.67	0.65
		SG1	0.92	0.79	0.92	0.58	0.92	0.79	0.79	0.54	0.54	0.75	0.75
		SG2	0.96	1.00	1.00	0.71	0.96	1.00	0.88	0.63	0.83	0.88	0.88
		NDSI	0.88	0.50	0.88	0.58	0.88	0.75	0.67	0.67	0.63	0.75	0.72

Supplemental Table 2. QTLs detected in the study

Trait	Chr	Position (cM)	Lod	Threshold[a]
P_V_PC10	1	100.0	3.7	3.5
P_A_PC6	1	186.0	5.6	3.7
B_N_PC7	1	187.1	4.9	3.5
B_A_PC6	1	188.0	4.6	3.6
B_V_PC2	2	216.0	4.4	3.7
P_A_PC9	3	0.0	4.8	4.4
PG	3	6.0	10.1	3.6
MW	3	6.0	11.2	4.9
B_A_PC1	3	8.0	4.2	3.6
B_N_PC1	3	8.0	4.1	3.8
B_V_PC5	7	98.0	4.4	3.5
B_A_PC9	7	98.0	6.9	5.0
B_A_PC10	7	108.0	3.8	3.4
B_A_PC6	8	0.0	3.6	3.6
PG	8	4.0	10.6	3.6
MW	8	5.3	13.2	4.9
DH	8	5.3	4.4	3.8
P_V_PC10	10	59.7	3.7	3.5
B_V_PC5	11	50.0	3.5	3.5
P_V_PC5	12	43.3	3.8	3.6

a. a threshold level of 5% for statistical significance was determined by 1000 permutations.
